# PHARYNGO-ILEO-COLO-ANASTOMOSIS WITH MICRO-VASCULAR BLOOD SUPPLY AUGMENTATION FOR ESOPHAGO-GASTRIC REPLACEMENT DUE TO ESOPHAGO-GASTRIC NECROSIS AFTER CAUSTIC INGESTION

**DOI:** 10.1590/0102-672020180001e1381

**Published:** 2018-07-02

**Authors:** Italo BRAGHETTO, Manuel FIGUEROA, Belén SANHUEZA, Enrique LANZARINI, Sergio SEPULVEDA, Christian ERAZO

**Affiliations:** 1 Gastrointestinal; 2 Microsurgery Unit, Department of Surgery, University Hospital Dr José J. Aguirre, Faculty of Medicine, University of Chile, Santiago, Chile.

**Keywords:** Necrosis. Esophagus, anastomosis, Surgery, ascending colon., Necrose. Esôfago, anastomose, Cirurgia, cólon ascendente.

## Abstract

*****Background*** ****:**

Complete esophago-gastric necrosis after caustic ingestion is a challenging surgical scenario for reconstruction of the upper digestive transit.

*****Aim*** ****:**

To present a surgical technique for reconstruction of the upper digestive tract after total esophagectomy and gastrectomy due to esophageal and gastric necrosis

***Method*:**

The transit was re-established by means of a pharyngo-ileo-colic interposition with microsurgical arterial and venous anastomosis for augmentation of blood supply. Colo-duodeno-anastomosis and ileo-transverse colic anastomosis were performed for complete digestive transit reconstruction.

***Result*:**

This procedure was applied in a case of 41 years male attempted suicide by ingesting alkali caustic liquid (concentrated sodium hydroxide). Total necrosis of the esophagus and stomach occurred, which required initially total esophago-gastrectomy, closure at the level of the crico-pharyngeal sphincter and jejunostomy for enteral feeding with a highly deteriorated quality of life***.*** The procedure was performed later and there were no major early and late postoperative complications and normal nutritional conditions were re-stablished.

***Conclusion*:**

The procedure is feasible and must be managed by multidisciplinary team in order to re-establish a normal quality of life.

## RESUMO

***Racional*:**

A necrose esofagogástrica completa após ingestão cáustica é um cenário cirúrgico desafiador para a reconstrução do trânsito digestivo alto.

***Objetivo*:**

Apresentar uma técnica cirúrgica para reconstrução do trato digestivo superior após esofagectomia total e gastrectomia por necrose esofágica e gástrica.

***Método*:**

O trânsito foi restabelecido por interposição faringo-íleo-cólica com anastomose arterial e venosa microcirúrgica com aumento do suprimento sanguíneo. Adicionalmente, anastomose colo-duodeno-anastomótica e íleo-transversa foram realizadas para reconstrução completa do trânsito digestivo.

***Resultado*:**

Este procedimento foi aplicado em um homem de 41 anos com tentativa de suicídio pela ingestão de líquido cáustico alcalino (hidróxido de sódio concentrado). Ocorreu necrose total do esôfago e do estômago, o que exigiu inicialmente esofagogastrectomia total, fechamento ao nível do esfíncter cricofaríngeo e jejunostomia para alimentação enteral produzindo qualidade de vida altamente deteriorada. O procedimento foi realizado mais tardiamente e não houve maiores complicações pós-operatórias precoces e tardias e condições nutricionais normais foram restabelecidas.

***Conclusão*:**

O procedimento é viável e deve ser manejado por equipe multidisciplinar a fim de restabelecer a qualidade de vida normal.

## INTRODUCTION

The reconstruction of the upper gastrointestinal transit is a surgical challenge^2^ ^,^ ^3^ ^,^ ^7^ After previous failed surgery and total disconnection of the digestive tract, colon interposition is used associated with high morbidity (50-70%)^4^ ^,^ ^5^. An additional difficulty occurs when there is no cervical esophagus available for reconstruction in patients with caustic ingestion. 

The aim of this study is to present a surgical technique to be used for reconstruction of the upper digestive transit with micro-vascular blood supply augmentation

## METHOD

This paper was conducted ethically in accordance with the World Medical Association Declaration of Helsinki.

In doing the procedure the patient must be attended by a multi-professional team, composed of gastrointestinal and micro-vascular surgeons, nutriologists, anesthesiologists, speech therapists, physiotherapists, endoscopists, and psychiatric support. 

### Surgical technique

**FIGURE 1:**
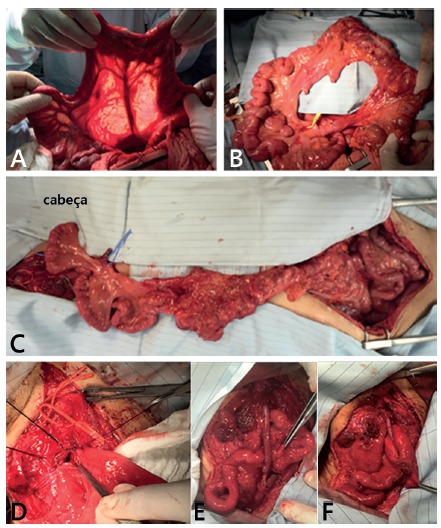
Intraoperative images of abdominal and cervical steps: A) transilumination of ileo-ceco-apendico-colic trunk; B) dissected right colon and distal ileum; C) ileocolon graft prepared for ascending; D) pharingo-ileo anastomosis in cervical step; E) vascular anastomosis F) final vision in a Tomoda loop fashion

A midline laparotomy, complete supra-infra mesocolic adhesiolysis is initially performed. Right colon and distal ileum are mobilized. Vascular pedicles are identified preserving right and middle colic vessels and ileal mesenteric vascular arcades in order to be used later for microvascular anastomosis. Sequencial surgical steps are: 1) ileo-ceco-apendico-colic trunk division and ligation at its origin and division of distal ileum at 20 cm before ileocecal valve; 2) transverse colon division left to the middle colic vessels with a lineal stapler violet cartridge (Medtronic^®^, Figure 1); 3) retrosternal tunnelization for isoperistaltic ileo-colon ascensus towards cervical region; 4) simultaneously, a left cervicotomy, dissection and identification of thyroid vessels, internal jugular vein and external carotid artery; 5) vascular pedicles are prepared; 6) ileum and right colon are rotated and ascended through the retrosternal tunnel toward the pharynx. Pharyngo-ileo anastomosis latero-lateral with Monocryl^®^ 3/0 interrupted manual sutures, tutored with a bougie 36F, and an ileo-ileal anastomosis in Tomoda fashion with Monocyl® 3/0 running suture are performed by digestive surgeons (Figure 1).

Augmentation of the arterial blood supply is obtained by arterial arcade pedicle of ileum to the superior thyroid artery and venous arcade pedicle to the lower thyroid vein with microvascular anastomosis with Ethylon^®^ 9/0 running suture. The procedure is completed with a manual 2-layer latero-lateral colo-duodenal anastomosis using Monocryl 3/0 and finally the ileo-transverse mechanic anastomosis with a Medtronic^®^ stapler white cartridge 60 mm is performed. A fine latex drain close to the duodenal anastomosis is exteriorized in the right upper abdominal quadrant and left in place. A cervical Penrose drain is placed. 

## RESULT

This procedure was applied in a case of 41 years male attempted suicide by ingesting alkali caustic liquid (concentrated sodium hydroxide). Total necrosis of the esophagus and stomach occurred, which required a total esophago-gastrectomy, closure at the level of the crico-pharyngeal sphincter and jejunostomy for enteral feeding. During the following three years, he presented a highly deteriorated quality of life, poor nutritional management and remained under psychiatric support (GIQLI score =73). The patient was evaluated confirming aphagia, emaciation (BMI=18.9) with severe protein calorie malnutrition, sarcopenia, (serum albumin=2.9), vitamin deficiency and anemia. Normal cardiopulmonary physical examination was observed.

**FIGURE 2:**
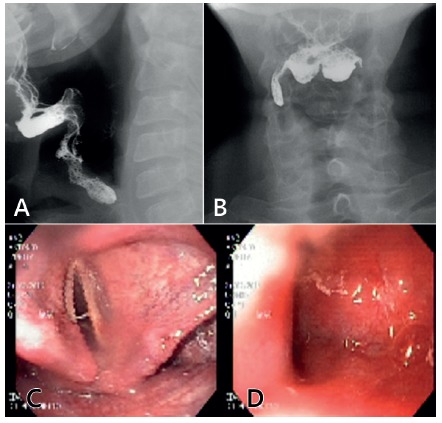
Preoperative evaluation - Barium swallow: A) lateral vision; B) frontal vision. Endoscopic studies: C and D) showing complete closure of pharynx

Barium sulphate swallow shows absence of passage of contrast distal to the pyriform sinus (Figure 2A and B), and endoscopy confirm radiological findings (Figure 2C and D). Barium enema and colonoscopy showed no pathological findings. CT angiography to assess the vascular supply of neck vessels, right colon and distal ileum demonstrated excellent pedicles and vascular arcades, with no pathological findings. 

The only alternative for surgical treatment was to perform colon interposition with pharyngo-ileo-colo anastomosis and surgical micro-vascular supplement to ensure blood supply as here described. Duration of surgery was 6 h and 55 min and total approximate bleeding was 250 ml. The patient was hospitalized for intensive medical nutrition and physical therapy. 

### Postoperative outcome

**FIGURE 3:**
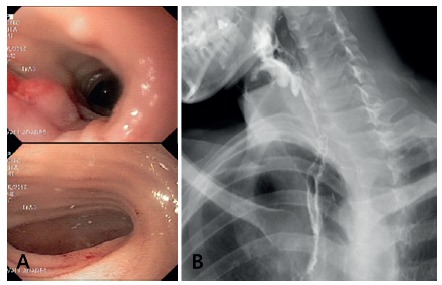
Postoperative control demonstrating absence of anastomotic stricture: A) postoperative endoscopy; B) barium swallow

Upper gastrointestinal bleeding with no hemodynamic repercussion or hemoglobin fall were observed most likely due to suture line bleeding of colo-duodenun anastomosis medically managed. No other complications were observed. The patient received initially total intravenous parenteral nutrition and enteral nutrition through jejunostomy. Oral water-soluble contrast medium swallow was indicated on the 7^th^ postoperative day which showed a mild stricture of the pharingo-ileo anastomosis, upper digestive endoscopy and dilatation with a Savary-Guillard 36F bougie were performed, obtaining very good transit to the ileo-colic segment (Figure 3A), endoscopuy, Figure 3B Barium swallow). After swallowing rehabilitation, oral ingestion was initiated. The patient was discharged on the 17^th^ postoperative day with oral and complementary enteral nutrition by jejunostomy. At the first post-operative month, he achieved complete oral nutrition and the jejunostomy was withdrawn.

### Follow-up

At the one year year follow-up, the patient continues with normal oral intake and supplementary vitamin treatment. An adequate nutritional status and a significantly improved quality of life were obtained, from a preoperative GIQLI score of 73 to a postoperative score of 122.

## DISCUSSION

There are occasions in which cervical esophagus is not even available and the anastomosis should be performed at the pharyngeal level^3,7^ being not possible to use the stomach and therefore a segment of colon is used. For this procedure, preferences have been described for different colic segments. The final decision on which segment to employ depends on the preference and experience of each surgical team^2,^ ^4^ ^,^ ^5^. In order to evaluate the type of colic graft to perform, a preoperative study with AngioTC 3-D of the abdomen and pelvis is very useful, which has 97.1% anatomical diagnostic accuracy of the mesenteric and colic vascular blood supply^8^. The rate of complications reported include necrosis of the flap (0-14%), anastomotic leaks (0-50%), anastomotic stenosis (0-32%), respiratory complications (10-42%), postoperative mortality (0-16.7%)^1^ ^,^ ^2^ ^,^ ^4^ ^,^ ^5^.

Recently, in order to assure an adequate blood supply of the ascending organ, several authors have reported cases using microsurgical anastomosis between the vessels of the ileal vascular arcade and vascular trunks of the neck with excellent results^6^. Considering these arguments, in order to prevent ischemia or necrosis of the graft, vascular blood supply potentialization was planned with microsurgical anastomosis to cervical vessels. Partial resection of the sternal manubrium and left clavicular head in order to have an adequate space for graft ascensus without compression of vascular irrigation sometime is needed^7^.

## CONCLUSION

These cases represent a very challenging clinical situation which must be evaluated and managed in a multidisciplinary and multi-professional way, in order to re-establish an almost normal quality of life. The procedure herein described is feasible to be used in cases of total caustic necrosis of esophagus and stomach, needing the resection of these segments in a prior operation.
